# Grey matter atrophy signatures and their genetic associations: a trans-disease study of Alzheimer’s disease and multiple sclerosis

**DOI:** 10.3389/fnimg.2026.1917609

**Published:** 2026-10-14

**Authors:** Yunfei Zhao, Jie Sun, Busharemu Reheman, Xinyi Bai, Che Zhang, Wenyu Ding, Bohong Zou, Han Zhang, Zhang Zhang, Ningnannan Zhang

**Affiliations:** 1 Department of Nuclear Medicine, Airport Hospital, Tianjin Medical University General Hospital, Tianjin, China; 2 Department of Radiology and Tianjin Key Laboratory of Functional Imaging, Tianjin Medical University General Hospital, Tianjin, China

**Keywords:** Allen Human Brain Atlas, Alzheimer disease, atrophy, Grey matter, meta-analysis, multiple sclerosis

## Abstract

**Background:**

Alzheimer’s disease (AD) and multiple sclerosis (MS) share neuroimmune mechanisms that drive neurodegeneration. Current clinical interventions are largely confined to a single-disease approach, owing to the absence of trans-disease diagnostic markers and common therapeutic targets.

**Objective:**

To map common and disease-specific grey matter (GM) atrophy patterns in AD and MS and identify their genetic correlations.

**Methods:**

We performed a trans-disease meta-analysis in large-sample neuroimaging data from 2001 to 2024 (2,070 MS versus 1,625 healthy control, and 1,624 AD versus 1,607 healthy control) to map GM atrophy regions. The cross-sample partial least squares regression was applied to integrate gene expression profiles from the Allen Human Brain Atlas with region-specific GM atrophy patterns, achieving cross-scale analysis of identify neurodegeneration-associated genes.

**Results:**

MS-specific atrophy localized to the bilateral dorsomedial thalamic nucleus and precentral gyrus, whereas AD-specific atrophy centered on bilateral hippocampus-amygdala complex in age-unadjusted comparisons. The common atrophy patterns were found in the bilateral superior temporal gyrus, insulae, posterior cingulate cortex, amygdalae, and striatal components. We identified 834 genes whose spatial expression patterns were associated with the common GM atrophy pattern in AD and MS, including PLS2 + and PLS2- subsets, which encompass biological processes such as immunological homeostasis imbalance, cellular stress response, and neurometabolic/functional regulation.

**Conclusion:**

Findings establish shared cortical–limbic-striatal atrophy as pivotal neurodegeneration nexus in AD and MS. The identified gene-imaging signatures provide insights into potential pathway-specific mechanisms and may inform future therapeutic target discovery for dual-disease intervention.

## Introduction

Alzheimer’s disease (AD) and multiple sclerosis (MS) are two prevalent neurological disorders affecting the central nervous system. With the rising life expectancy of MS patients, there is increased attention on ageing and age-related neurodegenerative diseases, such as AD (Luczynski et al., 2019; Filippi et al., 2024). Although there are fundamental differences in their epidemiology and pathobiology, AD and MS share some clinical and etiological characteristics (Lassmann, 2011; Louveau et al., 2016; Rossi et al., 2021). In AD, visuospatial deficits may emerge early, whereas older patients with MS may more commonly experience learning and memory impairments. These cognitive manifestations in MS may resemble those observed in AD (Oishi et al., 2020; Benedict et al., 2020; Barkhof et al., 1993). AD and MS share certain pathological features as documented in case reports and studies (Luczynski et al., 2019; Barkhof et al., 1993; Dal Bianco et al., 2008; Frischer et al., 2009; Flanagan et al., 2014; Zeydan et al., 2020; Kolanko et al., 2020; Kobylarek et al., 2020). For example, a postmortem study of 45 patients with MS, 9 patients with AD, and 15 controls found pronounced cortical microglial activation in both MS and AD, with closely similar activation patterns. AD-type pathology, including *β*-amyloid plaques and neurofibrillary tangles, was also identified in a subset of MS cases. In addition, a study of three patients with MS and progressive cognitive impairment identified amyloid positivity in two patients, who were subsequently diagnosed with coexisting AD. Recent evidence suggest that myelin damage in MS intersects with AD pathogenesis through shared molecular mechanisms (Sulthana et al., 2025; Sanadgol et al., 2025). Common factors, including environmental factors (e.g., viral infections like HSV-1), neuroinflammation, mitochondrial alterations, energy deficiencies, and dysregulation of cellular or lymphatic components, may contribute to both conditions (Lassmann, 2011; Louveau et al., 2016; Rossi et al., 2021; Boukhvalova et al., 2023; Stojic-Vukanic et al., 2020; Leszek et al., 2016; Abdelhak et al., 2022; Temmerman et al., 2023). The relationship between AD and MS is complex. Although much research has focused on the harmful effects of these mechanisms in AD and MS individually, recent research suggests that certain aspects of MS biology, or specific genetic factors in MS patients, may offer protection against AD (Brier et al., 2024), suggesting undiscovered neuroprotective crosstalk.

To explore the common mechanism of these two diseases, we can begin by examining the brain structures that are commonly affected by atrophy in both. Grey matter (GM) atrophy is a reliable biomarker for neurodegeneration in neuroimaging studies. In AD, volume reductions in the hippocampal and entorhinal cortex are noted and occur early, then gradually extend to other regions. In contrast, MS is characterized by more widespread and variable brain atrophy, with a preference for specific areas such as the thalamus and deep GM. The ability to detect regional and global atrophy, often before the onset of symptoms, makes GM atrophy a valuable tool for early diagnosis and monitoring disease progression (Jack et al., 2018; Wattjes et al., 2021). Identifying their common GM atrophy patterns and dissecting their transcriptomic correlates helps uncover the molecular basis of shared regional vulnerability under divergent etiologies, and explains why disorders with disparate pathological mechanisms give rise to similar brain-structural and cognitive changes. Meanwhile, cross-disease-validated shared imaging and molecular signatures offer new directions for developing transdiagnostic biomarkers of cognitive impairment and potential therapeutic targets with relevance across disorders. Additionally, several genetic loci associated with susceptibility to AD and MS have been identified separately through large-scale genome-wide association studies (GWAS) (Chia et al., 2021; International Multiple Sclerosis Genetics C, 2019). The recent identification of shared genetic loci between AD and MS further highlights the potential for comorbidity (Fominykh et al., 2023). Studies have also revealed a genetic influence on the etiology of both diseases, and some research has explored potential connections through specific genetic variant (Javor et al., 2022). Additionally, previous investigations have independently examined the relationship between gene expression and GM atrophy in each condition using cross-sample spatial correlations with genome-wide expression data from the Allen Human Brain Atlas (AHBA). These studies have provided valuable clues to the neurobiological mechanisms underlying GM atrophy in AD and MS (Hawrylycz et al., 2015; Sun et al., 2022; Groot et al., 2021).

Here, we conduct a trans-disease spatial transcriptomic-imaging atlas study, aiming to identify common and disease-specific patterns of GM atrophy in AD and MS through meta-analyses and subgroup conjunction analyses. Subsequently, partial least squares regression (PLSR) is applied to investigate association between gene expression profiles from the AHBA and the derived common GM volume maps. Finally, functional annotation was performed to elucidate the neurobiological mechanisms of genes whose spatial expression patterns were associated with the common GM atrophy pattern in both diseases.

## Materials and methods

The processing pipeline is illustrated schematically in Figure 1.

**Figure 1:**
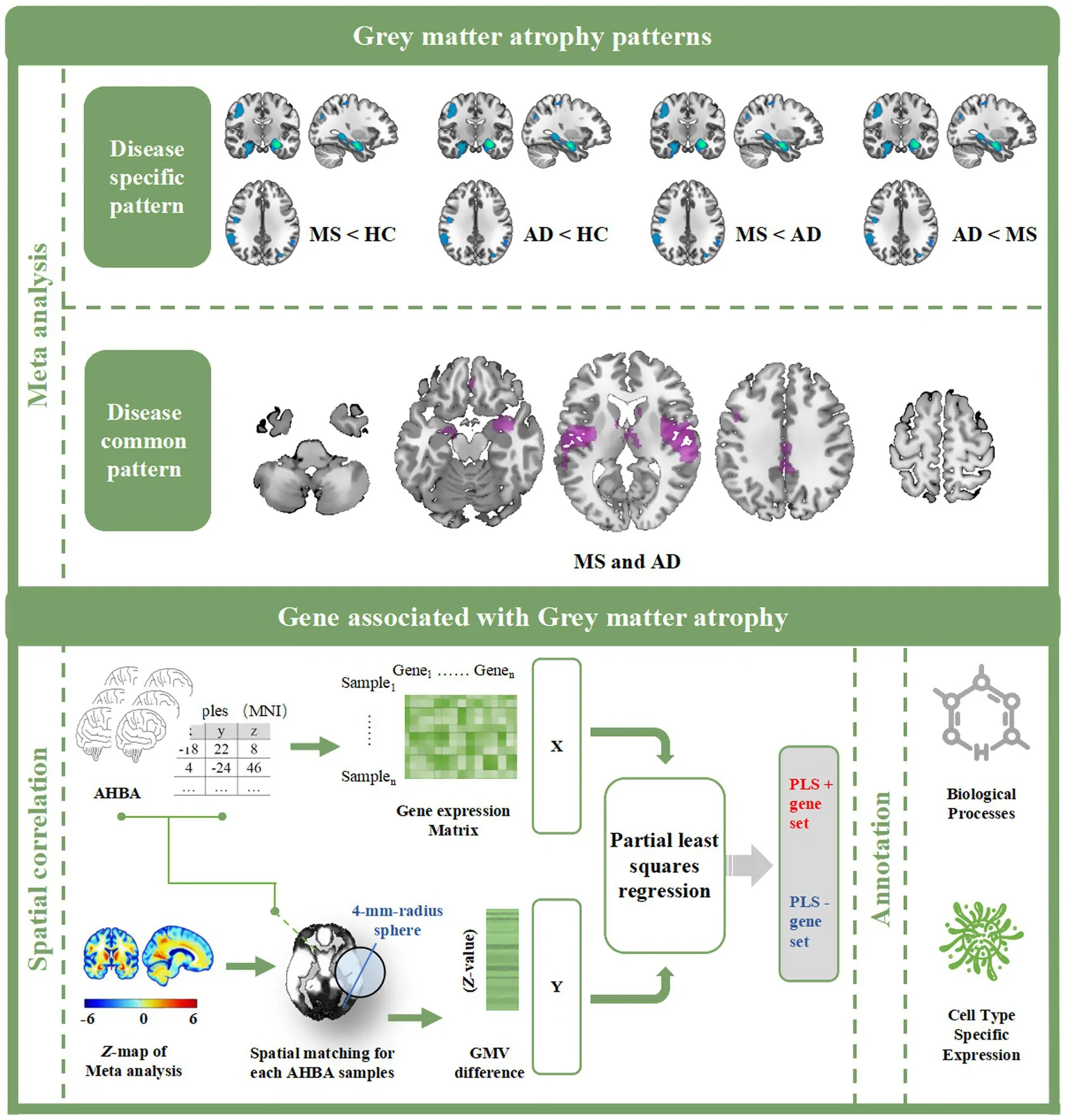
Flowchart of the study design. AD, Alzheimer’s disease; AHBA, Allen Human Brain Atlas; MS, multiple sclerosis; HC, healthy control.

### Standard protocol approvals, registrations, and patient consents

IRB approval and patient consent were not required due to the type of study, as all data used in this study were obtained from publicly available, de-identified databases. The systematic review and meta-analysis component of this study was not prospectively registered in PROSPERO.

### Search strategy

A comprehensive search was performed in PubMed from January 2001 to December 2024 with the following keywords: (1) (“multiple sclerosis” OR “MS”) AND (“voxel based morphometry” OR “VBM”) AND (“atrophy”) AND (“grey matter” OR “GM”); (2) (“Alzheimer’s disease” OR “AD”) AND (“voxel based morphometry” OR “VBM”) AND (“atrophy”) AND (“grey matter” OR “GM”).

### Selection criteria for meta-analysis

The inclusion criteria were: (1) the peak coordinates of significant GM clusters were reported in Talairach/Tournoux or Montreal Neurological Institute (MNI) coordinates; (2) the GM atrophy of the whole brain was assessed by VBM, one of the most prevalent techniques used to evaluate the cerebral GM atrophy; (3) comparison between patients (AD or MS) and healthy control (HC); (4) adult subjects with ages greater than 18 years.

The exclusion criteria were: (1) studies published before 2000 were excluded to reduce methodological heterogeneity in early VBM studies (Ashburner and Friston, 2000); (2) unreported coordinates or unavailable full text; (3) studies with fewer than nine participants were excluded because very small sample sizes may provide insufficient statistical power and yield less stable and less replicable neuroimaging findings (Turner et al., 2018; Radua et al., 2010; Pan et al., 2012); (4) the data overlapped with those of another publication; (5) studies limit their analyses to specific regions of interest (ROI); (6) non-independent analyses, such as those originating from the same center or involving duplicate subjects.

Two researchers (YZ and JS) read the title, abstract and main text of each article obtained from the initial search, and made independent inference based on the above inclusion and exclusion criteria. Discrepancy was resolved by a senior researcher (NZ), and the selection strategy is provided in Figure 2. PRISMA guidelines were followed as recommended in the study of Moher.

**Figure 2:**
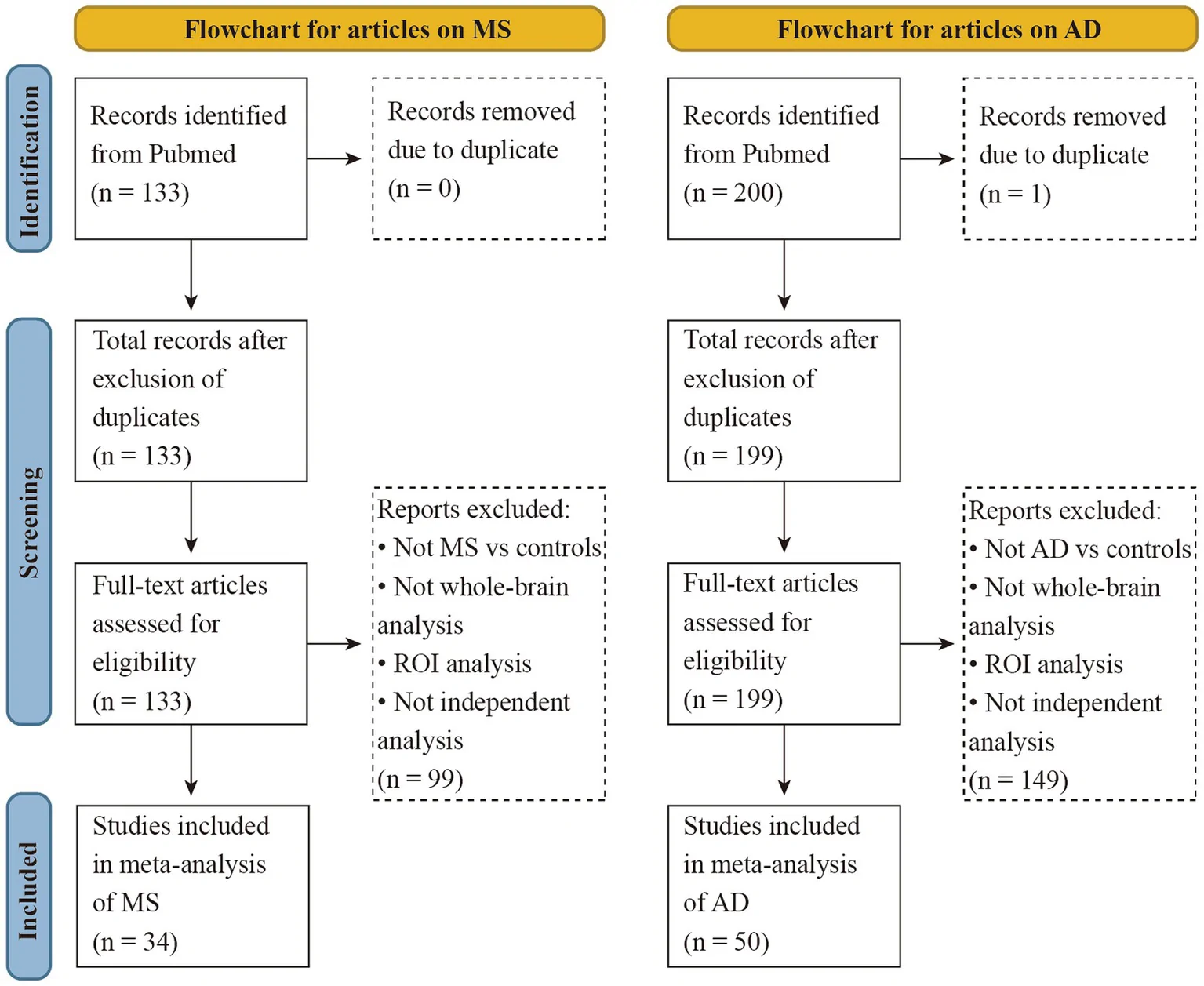
Flowcharts of the literature search and selection criteria for articles in the meta-analysis. AD, Alzheimer’s disease; MS, multiple sclerosis; HC, healthy control; ROI, region of interest.

### Meta-analysis process and bias analysis

We conducted a meta-analysis to investigate the GM atrophy in patients versus HC (i.e., AD versus HC, MS versus HC). Subsequently, a trans-disease meta-analysis was further carried out to assess the differences in GM atrophy between AD and MS using a linear model. Age was included as a covariate in the meta-regression analyses comparing AD and MS. The analyses were performed in a standard process using the Seed-based Mapping (SDM) v6.22.

The meta-analysis technique for neuroimaging studies was based on peak coordinates of the identified effects (Radua and Mataix-Cols, 2012). Talairach coordinates were converted into MNI space using the icbm2tal tools (Lancaster et al., 2007). We converted *Z* values or *p* values into *T*-statistics using the online converter. The full-width at half maximum in SDM were set at 20 mm, because in previous simulations it has been found to have an excellent control of false positives results in a preprocessing step. The proposed statistical thresholds were included as the following: uncorrected voxel-level *p* < 0.005, extent threshold of peak height *Z* ≥ 1, cluster extent ≥10 voxels (Radua et al., 2012). To improve the anatomical specificity of the identified GM clusters, the peak coordinates of significant clusters were assigned to cytoarchitectonic regions using the probabilistic maps of the Julich Brain Atlas using the SPM Anatomy Toolbox (Eickhoff et al., 2005). For each peak coordinate, the cytoarchitectonic area with the highest probability was identified and reported.

The robustness of the results was further assessed using cluster-level threshold-free cluster enhancement (TFCE) analysis with family-wise error (FWE) correction at *p* < 0.005 (Albajes-Eizagirre et al., 2019), systematic whole-brain voxel-based jackknife sensitivity analysis, and Egger’s regression test for publication bias (Egger et al., 1997). All above processes were referred to the SDM Tutorial^1^ and publications.

### Conjunction analysis and cognitive relevance image decoder

To identify the regions of common GM atrophy in AD and MS, subgroup contrasts of brain imaging were summarized in a meta-analytic map, and the overlapping *p* values of the GM atrophy maps were then computed (Radua et al., 2013). The method we implemented in SDM considered the underlying noise of the meta-analytic estimation. The voxel-level threshold for conjunction analysis was set by *p* < 0.0025.

To further understand the cognitive relevance of common GM atrophy in AD and MS, we conducted the cross-sample spatial correlation analyses between the *Z*-maps of GM atrophy and values in each brain activation map for cognitive domains using Neurosynth, a well-validated and publicly accessible platform for functional neuroimaging literature meta-analysis^2^ (Yarkoni et al., 2011). The Neurosynth database offers a variety of brain activation maps for specific neural processes, which we specifically selected based on cognition-related items from cognitive atlas^3^ (Poldrack et al., 2011).

### Spatial association of transcriptome and neuroimaging

A partial least squares regression (PLSR) model was used to identify gene-expression patterns spatially associated with the GM atrophy profile derived from the meta-analysis (Abdi, 2010). Brain gene expression data were extracted from the AHBA dataset^4^ (Hawrylycz et al., 2015; Sun et al., 2022). Gene expression data processing and the demographics of each donor are presented in supplement and Supplementary Table S1. To spatially link transcriptomic and neuroimaging data, a 4-mm-radius sphere was centered at the MNI coordinate of each AHBA tissue sample, and the mean *Z*-value within the sphere was extracted from the meta-analysis *Z* map. Thus, each tissue sample was characterized by both a gene-expression profile and a spatially corresponding GM *Z*-value. The standardized sample-by-gene expression matrix was entered into PLSR as the predictor matrix, with the corresponding standardized GM Z-values as the response variable. Five PLS components were estimated and ranked according to the variance explained in the GM atrophy profile. The component accounting for more than 10% of the variance and achieved statistical significance beyond the threshold of chance (permutation *p* < 0.05) was selected (Groot et al., 2021). Additionally, the bootstrapping technique was employed to assess the significance of genes contributing to components. The PLS weights of each gene were normalized by dividing the standard error from bootstrapping, and was utilized to rank the genes according to their PLS component contributions. Finally, only the significant genes (Bonferroni-corrected *p* < 0.05; *Z*-value with both positive and negative value) were all utilized for the following functional annotation analyses.

To tackle the spatial autocorrelation (SA), we conducted the permutation test by using a spatially constrained null model proposed by Burt et al. (Burt et al., 2018; Vos de Wael et al., 2020; Burt et al., 2020), which is carried out in a Python-based, open-source software application entitled BrainSMASH: Brain Surrogate Maps with Autocorrelated Spatial Heterogeneity^5^. BrainSMASH was used to generate surrogate GM maps that preserved the spatial autocorrelation structure of the empirical meta-analysis *Z* map. For each surrogate map, the empirical gene-expression matrix was held fixed, whereas the empirical GM *Z*-values were replaced by the surrogate *Z*-values, and the same PLSR procedure was repeated. We repeated this method for 1,000 times, and the variance explained by each PLS component was recorded to construct a spatial null distribution. The permutation *p* value was calculated as the proportion of surrogate models in which the explained variance of the corresponding PLS component exceeded that obtained from the empirical data.

### Gene set enrichment analysis

A variety of enrichment analyses were performed on the genes whose spatial expression patterns were associated with the common GM atrophy pattern in AD and MS. Firstly, we applied analyses from the gene category enrichment analysis (GCEA) toolbox to identify the biological processes (BP) in which the gene sets determined by PLSR are significantly involved (available at https://github.com/benfulcher/GeneCategoryEnrichmentAnalysis) (Fulcher et al., 2020). A spatial brain phenotype (SBP)-spatial ensemble null-distribution was constructed to reduce the SA-related false-positives bias. We specifically employed Gene Ontology (GO) term hierarchy and annotation files for *Homo sapiens* (data version: 2019-04-17), which were packed from GO^6^. Genes with both positive and negative *Z*-value were subjected to a separate enrichment analysis to determine the BP categories in which they are most involved. The gene loading was determined by calculating the *Spearman’s* correlation between the expression values of individual genes and the scores derived from PLSR, and mean gene loadings within each category were utilized to calculate the category score. A permutation test was performed on each GO category, adopting 1,000 SA-preserved permutations on *Z*-maps. The category score was utilized as the statistic to generate the null distribution. The resulting category score was compared to the null distribution to determine its significance, and a *p*-value <0.05 was judged statistically significant. We also performed Kyoto Encyclopedia of Genes and Genomes (KEGG) pathway enrichment analysis separately to provide pathway-level insights into the underlying molecular mechanisms (Kanehisa et al., 2021). KEGG pathways with an FDR-adjusted *q* value 0.05 were considered significantly enriched. Secondly, we conducted cell and region specific expression analyses by using online cell type-specific expression analysis (CSEA) tools^7^ (Dougherty et al., 2010). We reported significant cell types and regions that can pass Benjamini-Hochberg FDR correction (*q* < 0.05, BH-FDR corrected).

## Results

### Meta-analysis cohort composition

We included a total of 84 articles in the meta-analysis, with 34 articles related to MS (2,070 patients of MS and 1,625 HC subjects) and 50 articles related to AD (1,624 patients of AD and 1,607 HC subjects). The process of the PRISMA-compliant articles selection workflow in meta-analysis is shown in Figure 2. Comprehensive demographic and clinical characteristics of the included articles are cataloged in Supplementary Table S2 (MS) and Supplementary Table S3 (AD), including age, sex, sample size and clinical subtype.

### The GM atrophy in disease vs. healthy controls

Compared to HC, patients with MS showed extensive GM atrophy across bilateral thalamus, striatal (caudate nucleus and putamen), insula, posterolateral temporal cortex, sensorimotor cortex and cingulate cortex, with focal GM atrophy in the left calcarine fissure, right fusiform gyrus, and right rectus gyrus (peak coordinates, Table 1a; spatial distribution, Figure 3a). The right thalamus, left caudate, left posterior cingulate gyrus, and right postcentral gyrus remained significant after FWE correction (Supplementary Table S4); Cytoarchitectonic mapping further showed that the peak in the right postcentral gyrus was assigned to Area 3b with a probability of 63.7% according to the Jülich Brain Atlas.

**Table 1:** Peak coordinates and cytoarchitectonic distribution of brain regions with grey matter atrophy in MS and AD in meta-analysis.

Peak MNI Coordinates			SDM-Z value	*p*-value	Voxels Size	Macroanato-mical structure	Cytoarchitectonic peak
x	y	z					
(a) MS < HC							
12	−28	−2	−11.234	~0	8,886	Right thalamus	—
−14	18	6	−8.925	~0	8,572	Left caudate	—
−6	−22	46	−9.162	~0	3,160	Left posterior cingulate	—
40	−20	46	−8.059	~0	2,326	Right postcentral gyrus	Area 3b (63.7%)
4	38	−12	−4.936	4.17E-07	632	Right superior frontal gyrus	Area s32 (65.8%)
2	−70	16	−4.506	3.28E-06	626	Left calcarine fissure	hOc2 [V2] (29.9%)
6	18	22	−3.396	3.42E-04	147	Left anterior cingulate	Area 33 (33.5%)
20	−60	−12	−3.461	2.69E-04	105	Right fusiform gyrus	hOc3v [V3v] (13.2%)
(b) AD < HC							
20	−2	−22	−14.153	~0	11,443	Right parahippocampal gyrus	Amygdala (LB) (51.0%)
−24	−10	−24	−12.971	~0	10,979	Left hippocampus	CA1 (Hippocampus) (52.5%)
0	−52	30	−7.256	~0	2093	Left posterior cingulate gyrus	—
2	44	4	−6.981	~0	1,327	Right anterior cingulate	Area p24ab (72.9%)
(c) MS < AD							
40	−18	44	−4.418	5.01E-06	731	Right precentral gyrus	Area 3b (71.7%)
14	−26	2	−7.254	~0	471	Right thalamus	—
−16	−26	4	−5.539	~0	311	Left thalamus	—
−38	−24	58	−3.334	4.28E-04	145	Left precentral gyrus	Area 4a (58.8%)
−54	−26	38	−3.106	9.48E-04	28	Left supramarginal gyrus	Area PFt (IPL) (73.6%)
18	0	20	−2.975	1.47E-03	24	Right caudate	—
(d) AD < MS							
20	0	−26	−6.814	~0	1,203	Right amygdala	Amygdala (LB) (47.5%)
−22	−6	−24	−7.551	~0	1,103	Left hippocampus	Amygdala (LB) (63.0%)
−58	−52	22	−4.969	1.79E-07	542	Left angular gyrus	Area PGa (IPL) (27.9%)
58	−40	30	−4.536	4.77E-07	478	Right supramarginal gyrus	Area PFm (IPL) (50.0%)
32	−34	−6	−4.879	1.64E-04	193	Right hippocampus	CA1 (Hippocampus) (54.4%)
(e) Common grey matter atrophy in AD and MS							
52	−18	10	−6.465	~0	3,629	Right superior temporal gyrus	—
−46	−10	−2	−7.72	~0	2,610	Left insula	—
0	−40	46	−4.431	4.71E-06	588	Left posterior cingulate	—
2	38	−12	−4.342	7.03E-06	320	Right superior frontal gyrus	Area s32 (65.5%)

AD, Alzheimer’s disease; MS, multiple sclerosis; HC, healthy control; MNI, Montreal Neurological Institute; “—” indicates that no corresponding cytoarchitectonic area was identified in the Jülich Brain Atlas at the peak coordinate.

References to cytoarchitectonic maps: amygdala (LB) (Amunts et al., 2005; Kedo et al., 2018); area 33 (Palomero-Gallagher et al., 2015); area 3b (Geyer et al., 2000); area 4a (Geyer et al., 1996); area p24ab (Palomero-Gallagher et al., 2019); area PFm (IPL) (Caspers et al., 2006; Caspers et al., 2008); area PFt (IPL) (Caspers et al., 2006; Caspers et al., 2008); area PGa (IPL) (Caspers et al., 2006; Caspers et al., 2008); area s32 (Palomero-Gallagher et al., 2015); CA1 (Hippocampus) (Amunts et al., 2005); hOc2 [V2] (Amunts et al., 2000); hOc3v [V3v] (Rottschy et al., 2007).

**Figure 3:**
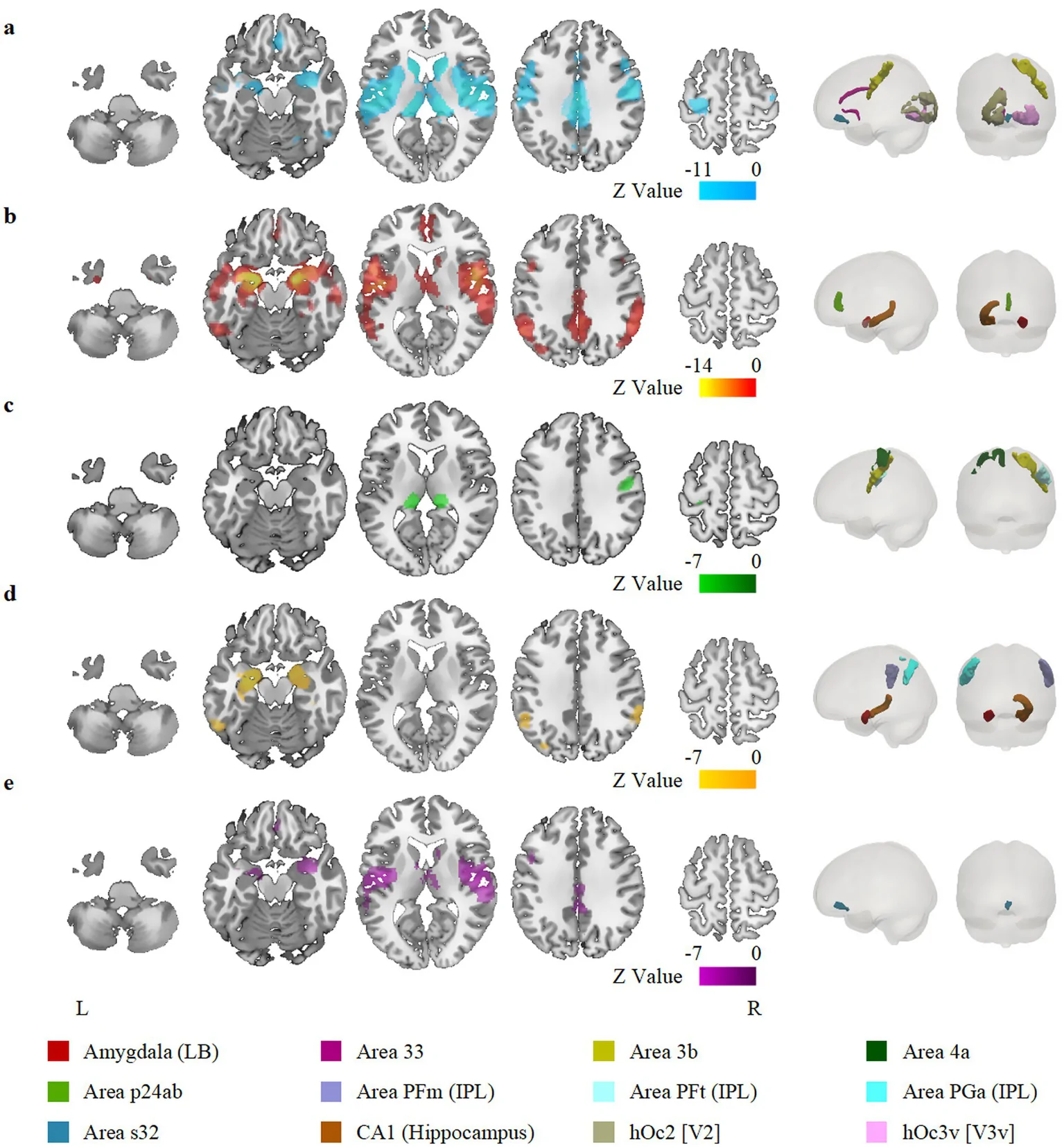
Results of Meta-analysis. Regions of GM atrophy and their cytoarchitectonic localization in **(a)** MS, compared to HC; **(b)** AD, compared to HC; **(c)** MS, compared to AD; **(d)** AD, compared to MS; and **(e)** both AD and MS, compared to HC. The voxel-wise statistical threshold is set as *p* = 0.005. The three-dimensional brain renderings on the right show the cytoarchitectonic regions assigned to the significant peak coordinates according to the probabilistic cytoarchitectonic maps of the Jülich Brain Atlas. Different colors represent different cytoarchitectonically defined regions, as indicated in the legend. Only peaks with identifiable cytoarchitectonic assignments in the Jülich Brain Atlas are displayed. AD, Alzheimer’s disease; MS, multiple sclerosis; GM, grey matter; HC, healthy control.

Compared to HC, patients with AD showed extensive GM atrophy as well, including bilateral medial temporal (hippocampus and parahippocampal gyrus), posterior cingulate cortex, insula, medial and posterolateral temporal cortex, supramarginal gyrus and angular gyrus and superior frontal gyrus, with focal GM atrophy in bilateral anterior thalamus and striatal (left putamen and right caudate nucleus) (peak coordinates, Table 1b; spatial distribution, Figure 3b). The right parahippocampal gyrus and left hippocampus remained significant after FWE correction (Supplementary Table S5). Cytoarchitectonic mapping further assigned the left hippocampal peak to the CA1 subfield with a probability of 52.5%, while the peak in the right parahippocampal gyrus showed a 51.0% probability of assignment to the laterobasal group of the amygdala (LB).

All findings demonstrated robust replicability through jackknife sensitivity analysis and null publication bias (MS vs. HC, Supplementary Table S6; AD vs. HC, Supplementary Table S7; Egger’s test *p* > 0.05).

### The disease-specific GM atrophy pattern

In the age-unadjusted analysis, compared to AD, patients of MS exhibited GM atrophy particularly in the bilateral dorsomedial thalamic nucleus and bilateral precentral gyrus (peak coordinates, Table 1c; spatial distribution, Figure 3c). At the cytoarchitectonic level, the right precentral peak showed a 71.7% probability of assignment to Area 3b, whereas the left precentral peak was assigned to Area 4a with a probability of 58.8%. Whereas, patients with AD showed preferential GM atrophy in the bilateral hippocampus, bilateral amygdala, left fusiform gyrus, left angular gyrus and right supramarginal gyrus relative to MS (peak coordinates, Table 1d; spatial distribution, Figure 3d). Cytoarchitectonic mapping further identified the laterobasal amygdala (LB; 47.5%) at the right amygdala peak and hippocampal CA1 (54.4%) at the right hippocampal peak. Adjusted for age, compared to AD, patients with MS showed greater GM loss in the right precentral gyrus and left postcentral gyrus. AD patients exhibited greater GM loss in the left supramarginal gyrus compared with MS patients (Supplementary Table S8).

### The common GM atrophy pattern

The overlapping GM atrophy regions in both AD and MS were observed in cortical regions, including bilateral superior temporal gyrus, bilateral insula, bilateral posterior cingulate cortex, and right rectus gyrus, as well as subcortical nuclei, including bilateral amygdala and striatum (left putamen and bilateral caudate nucleus) (peak coordinates, Table 1e; spatial distribution, Figure 3e). For the shared GM atrophy pattern, the peak macroanatomically labeled as the right superior frontal gyrus showed a 65.5% probability of assignment to cytoarchitectonic Area s32 in the Jülich Brain Atlas.

### Neurosynth decoding of common GM atrophy pattern

By connecting GM atrophy maps with brain activation maps of cognitive terms in Neurosynth, the top 20 terms with highest *r*-value were selected (Figure 4a). These cognitive concepts delineate a trans modal cognitive architecture encompassing speech production, perceptual integration (auditory processing, face recognition), affective-somatic regulation (pain perception, emotional regulation), motor-cognitive interfaces (movements, decision making).

**Figure 4:**
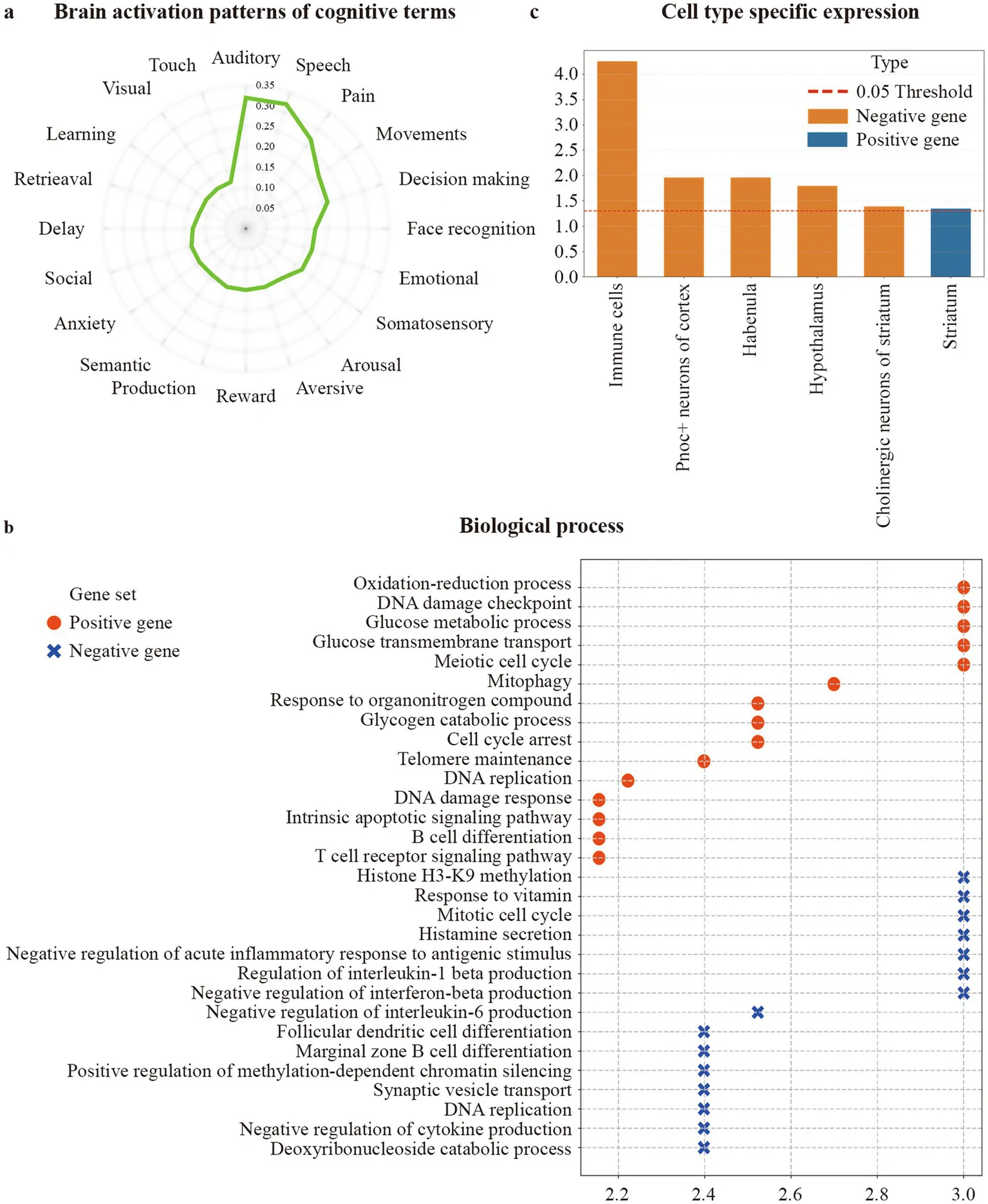
Gene enrichment analyses associated with common GM atrophy pattern in AD and MS. **(a)** Neurosynth decoding of GM atrophy. The top 20 cognitive terms correlated with the common GM atrophy pattern in AD and MS. **(b)** The top 15 terms that show significant enrichment in the biological process for both the positive and negative gene sets. The x-axis shows the -log_10_p value for each biological process item (y-axis). Red dots represent the results of genes in positive set, while blue ones represent the results of genes in negative set. **(c)** Results of the cell type-specific expression analysis. The y-axis indicates -log_10_ *p*-value and the x-axis indicates cell types. Orange bars represent genes in negative set, while blue bar represents gene in positive set. AD, Alzheimer’s disease; MS, multiple sclerosis; GM, grey matter.

### Genes corelated with common GM atrophy pattern

PLS1 and PLS2 explained 13.76 and 13.10% of the variance, respectively. However, only PLS2 reached significance in the spatial autocorrelation preserving permutation test (PLS1, *p* = 0.402; PLS2, *p* = 0.019); therefore, PLS2 was retained for subsequent analyses. The PLS2 score exhibited a spatially significant positive correlation with *Z*-scores of overlapping GM atrophy regions (*Spearman’s r* = 0.37, *p* = 0.001, permutation test with SA corrected, 1,000 permutations, Supplementary Figure S1). Subsequently, we identified a total of 834 genes expression significantly associated with common GM atrophy in AD and MS, through the analysis of spatial correlation between the transcriptome and neuroimaging. These genes were then categorized into two sets based on their standardized weights in PLS2: a positive set including 290 genes (PLS2 +; *Z*-scores >0) and a negative set including 544 genes (PLS2 -; *Z*-scores <0).

### Enrichment analysis

The genes whose spatial expression patterns were associated with the common GM atrophy pattern in AD and MS showed exploratory GO enrichment patterns in several BPs. For the positive set, genes showed significant functional enrichment in 89 terms of BPs (uncorrected *p* < 0.05; Supplementary Table S9), mainly in cellular and molecular processes, DNA repair, apoptosis, and cell survival, immune response and signaling pathways, development and morphogenesis, protein and cellular dynamics (Figure 4b); and significantly expressed in striatum (*q* < 0.05, FDR correction; Figure 4c; Supplementary Table S11). For the negative set, genes were significantly enriched for 101 terms of BPs (*p* < 0.05; Supplementary Table S10), mainly in epigenetic regulation and chromatin modifications, vitamin metabolism, immune response and inflammation regulation, development and differentiation, metabolism and cellular processes, cellular signaling and response to stimuli, neurological and sensory processes, stress responses and cell survival, immune and inflammatory responses, development and morphogenesis (Figure 4b); and specifically expressed in immune cells, Pnoc+ neurons of cortex, habenula, and hypothalamus (*q* < 0.05, FDR correction; Figure 4c; Supplementary Table S12). To complement the GO enrichment results, we additionally performed KEGG pathway enrichment analysis (*q* < 0.05, FDR correction, Supplementary Figure S2). The two analyses exhibited biological consistency and converged on four core themes: neurodegenerative injury, immune-inflammatory regulation, energy metabolic homeostasis, and synaptic-axonal maintenance.

## Discussion

### Common atrophy regions and trans-disease cognitive domain-specific

The common GM atrophy regions of AD and MS is reported in our study in the bilateral superior temporal gyri, posterior cingulate cortices, amygdalae, insulae, right rectus gyrus, and striatum. Most regions are key components of the auditory/language network and the emotion processing network. The disruption of these networks’ circuits due to structural degeneration constitutes the common neural substrate underlying trans-disease cognitive impairments in both conditions.

Within the language/auditory network, the superior temporal gyrus exhibits distinct injury pathways yet result in disrupting auditory-verbal processing circuitry. As the primary hub region for auditory signal processing, superior temporal gyrus atrophy directly compromises auditory information integrity in both diseases. For example, MS lesions within auditory pathways cause neural signal conduction dysfunction, manifesting as sensorineural hearing loss (Cruz et al., 2022) while in AD, neuronal loss and functional abnormalities (e.g., altered spontaneous low-frequency activity) impair semantic encoding, leading to auditory-verbal learning deficits (Gao et al., 2023).

Within the emotional processing network, a core circuit comprises the bilateral amygdala, insula, orbitofrontal cortex, and striatum. The structural changes in these regions consistently impair emotion recognition and regulation in both AD and MS. Critically, amygdalae atrophy demonstrates trans-disease consistency: it predicts fear/anger recognition deficits in MS (Hu et al., 2023) and correlates with emotional processing impairments in AD (Kumfor et al., 2014). This association between structural damage and impaired negative emotion recognition reflects the functional conservation of this brain region in core emotional encoding. The orbitofrontal cortex dysfunction further exacerbates emotional disturbances. MS patients exhibit greater variability in left orbitofrontal cortex responses to positive emotional stimuli compared with control subjects (Mike et al., 2013). In AD, orbitofrontal cortex tau deposition induces local neurotoxicity and cortical atrophy, significantly contributing to apathy (Kitamura et al., 2018). As a synergistic regulator, striatal volume reduction associates with depression risk in MS (Hu et al., 2023), while its atrophy contributes to emotional processing deficits in AD (Kitamura et al., 2018). This confirms the striatum’s role as a pivotal supplementary node within the emotional circuit.

### Disease-specific pathology-driven differences in common atrophy pattern and their clinical utility

It is worth noting that the pathology-driven neural basis of common atrophy region varies depending on the nature of the disease. Additionally, some regional differences identified in the unadjusted analysis, particularly those involving the thalamic and hippocampal-amygdala regions, were attenuated after age adjustment, suggesting that age contributes to part of the observed between-disease variation. On one hand, pathology-driven differentiation in injury mechanisms. For example (Cruz et al., 2022; Gao et al., 2023), both conditions cause superior temporal gyrus atrophy, but through distinct pathological mechanisms: MS via inflammatory demyelinating damage further disrupts higher-order processing of auditory information, whereas AD-related neuronal dysfunction and cortical atrophy are more closely associated with tau deposition (Gordon et al., 2018; Xia et al., 2017). Subsequently, superior temporal gyrus atrophy demonstrates association with clinical phenotypes of sensorineural hearing loss in MS and verbal learning impairment in AD. On the other hand, MS and AD may involve different patterns of neural circuit reorganization due to their distinct pathological substrates. In MS, inflammatory demyelination and neurodegenerative processes may lead to dynamic alterations in functional and structural brain networks. Longitudinal MRI studies have demonstrated that changes in functional connectivity and brain structure are associated with cognitive trajectories in MS patients (Azzimonti et al., 2023). Moreover, the insula, as a key node of the salience network involved in interoception, emotional processing, and network switching, has been implicated in neurodegenerative disorders (Hazelton et al., 2025). These network alterations may reflect adaptive reorganization or disease-related disruption. In contrast, AD-related functional connectivity alterations occur within the context of progressive Aβ/Tau pathology and neuronal loss. Such alterations may reflect network dysfunction (Corriveau-Lecavalier et al., 2026), increased cognitive demands and cascading network failure (Jones et al., 2016), or pathological processes facilitating tau propagation through connected brain regions (Roemer-Cassiano et al., 2025).

These findings offer significant implications for clinical translation. Superior temporal gyrus atrophy may be associated with auditory and linguistic dysfunction in MS and AD (Harasty et al., 1999; Stellmann et al., 2021), although the functional significance of this structural alteration requires further validation using domain-specific behavioral measures. Neuro-regulation targeting the amygdala, insula, orbitofrontal cortex, and striatum circuit (such as transcranial magnetic stimulation) may simultaneously improve emotional symptoms in both disorders.

### The genetic association

In our study, PLSR identified a gene-expression pattern whose spatial distribution across brain regions was associated with the spatial pattern of GM atrophy. Genes with significant positive or negative weights on the selected PLS component were classified as positive- and negative-weighted genes, respectively. Thus, the positive and negative weights indicate that these genes contribute in different directions to the PLS-derived gene-expression pattern associated with GM atrophy.

The PLS2 + gene set predominantly converges on how cell respond to internal/external stress (particularly to genomic damage and metabolic/energy challenges), and determines cellular fate (repair, survival, or death) by coordinating mechanisms such as DNA stability maintenance, cell cycle arrest, metabolic remodeling, and quality control (mitophagy/apoptosis). In common atrophy regions of AD and MS, failure of these defenses accelerates neural degeneration (neurons, axons, synapses) and induces severe mitochondrial dysfunction. Axonal energy deficits from mitochondrial impairment disrupt transport and promote cell death (Dutta et al., 2006; Trapp and Stys, 2009). In oligodendrocytes, mitochondrial damage is linked to apoptosis, which is mediated by the release of apoptosis-inducing factors from mitochondrial stores (Trapp and Stys, 2009; Veto et al., 2010). Reduced mitochondrial density in AD indicates an oxidative stress burden (Santos et al., 2013). Autophagic/lysosomal accumulation of damaged mitochondrial components (mitochondrial DNA abnormalities, impaired cytochrome c oxidase activity, often with lipofuscin) compromises cellular viability (Hirai et al., 2001; Moreira et al., 2010). Notably, PLS2 + genes also show significant enrichment in adaptive immune cell differentiation/activation pathways (B/T cells), suggesting these core stress/steady-state mechanisms regulate physiological functions in specialized cell populations. The core pathology of MS is autoimmune inflammation-mediated myelin destruction, while PLS2 + gene-enriched immune cell activation and migration pathways (e.g., T-cell receptor signaling, cytoskeletal reorganization) are key drivers of inflammation. For example, T cell migration across the blood–brain barrier is a precondition for MS lesion formation (von Essen et al., 2025). Inhibiting this process directly reduces immune cell infiltration and alleviates demyelination injury (Borjini et al., 2025).

Conversely, PLS2- genes are functionally characterized by negative regulation of immune responses (particularly inflammation), specification of immune cell subsets, such as marginal zone B cells and follicular dendritic cells and maintaining fundamental processes like cellular proliferation and genetic material stability (e.g., DNA replication). Its functional characteristics are manifested in actively suppressing excessive inflammation, such as inhibition of interleukin-1β, interferon-β, and interleukin-6 production, shaping immune microenvironments, maintaining basic cell functions, and achieving long-term regulation through epigenetic modifications (e.g., positive regulation of methylation-dependent chromatin silencing) and regulated secretion/transport (e.g., synaptic vesicle transport).

## Limitations

Several limitations should be noted. First, the spatial correlation analysis used postmortem gene expression data from AHBA and neuroimaging data from meta-analysis *Z*-maps. While this approach can identify genes with conserved expression patterns (Hawrylycz et al., 2015), it may miss those with significant interindividual variability. Therefore, the observed associations should be interpreted as spatial relationships between normative gene-expression patterns and disease-related GM atrophy, rather than as evidence of differential gene expression in patients or direct causal effects of the identified genes on GM atrophy. Second, MS clinical subtypes exhibit distinct neurobiological features, and their genetic heterogeneity could influence our results. Third, the typical onset age differs substantially between MS (20–40 years) and AD (≥65 years), potentially confounding our findings. Finally, because AD and MS have distinct primary neuropathological mechanisms, the shared GM atrophy regions and transcriptomic patterns identified through this trans-disease analysis in our study may represent phenotypic outcomes induced by different disease processes, rather than evidence of shared pathogenic mechanism between AD and MS. Furthermore, our findings cannot be used for clinical diagnosis. AD and MS have their respective independent clinical diagnostic criteria, and our results cannot replace the existing diagnostic systems.

## Conclusion

This study presents neuroimaging-genetic framework in trans-disease cohort. Our findings provide a comprehensive meta-analysis of neuroimaging data in AD and MS, and identify shared cortical–limbic-striatal atrophy as the key neurodegeneration nexus structures.

## Data Availability

The original contributions presented in the study are included in the article/Supplementary material, further inquiries can be directed to the corresponding authors.

## Footnotes

1
http://sdmproject.com/software/Tutorial

2
https://neurosynth.org/

3
http://www.cognitiveatlas.org/

4
http://www.brain-map.org

5
https://github.com/murraylab/brainsmash

6
http://geneontology.org/

7
http://doughertytools.wustl.edu/CSEAtool.html
