# Supplementary material for: Grey matter atrophy signatures and their genetic associations: a trans-disease study of Alzheimer’s disease and multiple sclerosis
Source: Front Neuroimaging. 2026 Oct 14;5:1917609. doi: 10.3389/fnimg.2026.1917609 (PMC13713765; doi:10.3389/fnimg.2026.1917609)
Supplement: Supplementary file 1 [file Data_sheet_1.docx]

**Supplementary Material**

[1. Supplementary Note	2](#_Toc238593572)

[1.1 Gene expression data preprocessing	2](#_Toc238593573)

[1.2 Gene expression data processing	2](#_Toc238593574)

[2. Supplementary Tables	3](#_Toc238593575)

[Table S1. The demographic information of each donor in AHBA dataset.	3](#_Toc238593576)

[Table S2. Detailed demographic and clinical characteristics of the 34 datasets related to MS included in the meta-analysis.	4](#_Toc238593577)

[Table S3. Detailed demographic and clinical characteristics of the 50 datasets related to AD included in the meta-analysis.	8](#_Toc238593578)

[Table S4. FWE-corrected sensitivity analysis of grey matter atrophy in MS compared with HC.	15](#_Toc238593579)

[Table S5. FWE-corrected sensitivity analysis of grey matter atrophy in AD compared with HC.	16](#_Toc238593580)

[Table S6. Egger’s test and Jackknife test of each peak in MS and HC subgroup meta-analysis.	17](#_Toc238593581)

[Table S7. Egger’s test and Jackknife test of each peak in AD and HC subgroup meta-analysis.	18](#_Toc238593582)

[Table S8. Age-adjusted meta-regression results for grey matter differences between MS and AD	19](#_Toc238593583)

[Table S9. Enrichment terms of the BP related to common positive gene of MS and AD.	20](#_Toc238593584)

[Table S10. Enrichment terms of the BP related to common negative gene of MS and AD.	24](#_Toc238593585)

[Table S11. The cell types that PLS2+ gene sets were expressed derived from specific expression analysis.	28](#_Toc238593586)

[Table S12. The cell types that PLS2- gene sets were expressed derived from specific expression analysis	29](#_Toc238593587)

[3. Supplementary Figures	30](#_Toc238593588)

[4. References	32](#_Toc238593589)

**1. Supplementary Note**

**1.1 Gene expression data preprocessing**

Firstly, we reannotated the genetic probes and retained only those that could be reliably matched to genes. These selected probes were then filtered based on their values in relation to background noise, employing a threshold of 50% [1]. Secondly, we removed probes that did not outperform background noise in at least 50% samples. Thirdly, we eliminate probes with weak correlation (Spearman rho < 0.2) with gene expression values measured by RNA-seq, and select the probe for a gene with the highest correlation to the RNA-seq data if two or more probes were saved. Fourthly, for the process of matching tissue samples with anatomical regions, the MNI coordinates of tissue samples from all six donors were adjusted using the alleninf package. Fifthly, we addressed the issue of donor-specific effects by using scaled robust sigmoid (SRS) normalization, to ensure that expression values are uniformly scaled across all donors, thereby mitigating any potential biases arising from donor-specific variations.

1.2 Gene expression data processing

Brain gene expression data were extracted from the AHBA dataset (http://www.brain-map.org) [2,3]. This dataset provides normalized microarray gene expression data for six human brains donated for study [3]. Four donors only had gene expression data for the left hemisphere, while two donors have gene expression data for the whole-brain. The demographics of each donor are presented in Table S1. A total of 3702 tissue samples from the cortex, subcortex, cerebellum, and brainstem were collected. Using custom 64K Agilent microarrays, the expression of over 20,000 genes in 3702 spatially distinct brain tissue samples was measured [2,3]. The AHBA gene expression data were preprocessed using abagen toolbox (https://www.github.com/netneurolab/abagen) [4]. Finally, these processing procedures generated normalized expression data of 15,633 genes for 3466 tissue samples.

**2. Supplementary Tables**

| **Table S1. The demographic information of each donor in AHBA dataset.** | | | |
| --- | --- | --- | --- |
| Donor ID | Age | Hemisphere |  |
| H0351.2001 | 20-29 | Left/Right |  |
| H0351.2002 | 30-39 | Left/Right |  |
| H0351.1009 | 50-59 | Left |  |
| H0351.1012 | 30-39 | Left |  |
| H0351.1015 | 40-49 | Left |  |
| H0351.1016 | 50-59 | Left |  |

*Note:* Data are publicly available from the AHBA [3].

**Table S2. Detailed demographic and clinical characteristics of the 34 datasets related to MS included in the meta-analysis.**

| Study |  | MS | | | | |  | HC | | | Analysis parameter | | |
| --- | --- | --- | --- | --- | --- | --- | --- | --- | --- | --- | --- | --- | --- |
| Year | Authors | Subtype | Sample size | M/F | Age^a^ (year) | Disease duration^a^ (year) |  | Sample size | M/F | Age^a^ (year) | Analysis software | Standard template | MRI field strength |
| 2006 | Morgen K. | RRMS | 19 | - | 32.4 | 1.6 |  | 19 | - | 31.7 | SPM2 | MNI | 1.5 T |
| 2007 | Khaleeli Z. | PPMS | 46 | 27/19 | 43.5 | 3.3 |  | 23 | 11/12 | 35.1 | SPM2 | MNI | 1.5 T |
| 2008 | Ceccarelli A. | BMS | 19 | 4/15 | 41.5 | - |  | 20 | 7/13 | 36.8 | SPM2 | MNI | 3.0 T |
|  | Ceccarelli A. | RRMS | 15 | 3/12 | 33.3 | - |  | 20 | 7/13 | 36.8 | SPM2 | MNI | 3.0 T |
| 2008 | Mesaros S. | BMS | 60 | 23/37 | 46.2 | 22.7 |  | 21 | 10/11 | 45.7 | - | MNI | 1.5 T |
|  | Mesaros S. | SPMS | 35 | 10/25 | 46.5 | 16.2 |  | 21 | 10/11 | 45.7 | - | MNI | 1.5 T |
| 2009 | Ceccarelli A. | PPMS | 18 | 8/10 | 49.6 | 10.7 |  | 17 | 6/11 | 51.3 | SPM2 | MNI | 3.0 T |
| 2009 | Bodini B. | PPMS | 35 | 20/15 | 44.8 | 3.3 |  | 23 | 11/12 | 35.1 | SPM2 | MNI | 1.5 T |
| 2010 | Prinster A. | RRMS | 128 | 42/86 | 36.1 | 10.1 |  | 35 | 17/18 | 38.3 | SPM5 | MNI | 1.5 T |
| 2010 | Spano B. | BMS | 10 | 2/8 | 44.5 | 17.1 |  | 20 | 8/12 | 40.5 | SPM5 | MNI | 3.0 T |
| 2010 | Riccitelli G. | Fa-RRMS | 10 | 4/6 | 38 | 8.2 |  | 14 | 8/6 | 38.7 | SPM5 | MNI | 1.5 T |
|  | Riccitelli G. | nFa-RRMS | 14 | 6/8 | 38.6 | 10.6 |  | 14 | 8/6 | 38.7 | SPM5 | MNI | 1.5 T |
| 2010 | Prakash R.S. | RRMS | 21 | 0/21 | 44.2 | 7.3 |  | 15 | 0/15 | 45.8 | FSL | MNI | 3.0 T |
| 2011 | Bonavita S. | RRMS | 36 | 15/21 | 40.7 | 11.4 |  | 18 | 8/10 | 39.0 | SPM8 | Talairach | 3.0 T |
| 2012 | Riccitelli G. | RRMS | 78 | 23/55 | 40.2 | 9.6 |  | 88 | 37/51 | 39.7 | SPM8 | MNI | 3.0 T |
| 2013 | Lin A. | RRMS | 11 | 4/7 | 38.5 | 30.5 |  | 11 | 4/7 | 39.5 | SPM8 | MNI | 3.0 T |
| 2013 | Cruz Gómez Á.J | Fa-RRMS | 32 | 11/21 | 37.7 | 7.4 |  | 18 | 10/8 | 31.1 | SPM8 | MNI | 1.5 T |
|  | Cruz Gómez Á.J | nFa-RRMS | 28 | 10/18 | 35 | 5.1 |  | 18 | 10/8 | 31.1 | SPM8 | MNI | 1.5 T |
| 2013 | Cerasa A. | Ce-RRMS | 12 | 2/10 | 38.9 | 12.1 |  | 20 | 6/14 | 36.9 | SPM8 | MNI | 1.5 T |
|  | Cerasa A. | nCe-RRMS | 14 | 3/11 | 38.6 | 8.8 |  | 20 | 6/14 | 36.9 | SPM8 | MNI | 1.5 T |
| 2014 | Gobbi C. | RRMS/BMS/PPMS | 123 | 52/71 | 41.7 | 12.6 |  | 90 | 39/51 | 39.7 | SPM8 | MNI | 3.0 T |
| 2014 | Tavazzi E. | PPMS | 18 | 12/6 | 46.9 | 12.4 |  | 31 | 11/20 | 47.9 | FSL | MNI | 1.5 T |
| 2014 | Parisi L. | C-MS | 9 | 2/7 | 50.2 | - |  | 9 | 3/6 | 54.4 | SPM8 | MNI | 1.5 T |
|  | Parisi L. | Cort-MS | 9 | 2/7 | 48.9 | - |  | 9 | 3/6 | 54.4 | SPM8 | MNI | 1.5 T |
| 2014 | Rocca M.A. | Fa-MS | 31 | 14/17 | 41 | 13.0 |  | 35 | 16/19 | 40.7 | FSL | MNI | 3.0 T |
|  | Rocca M.A. | nFa-MS | 32 | 15/17 | 39.5 | 11.7 |  | 35 | 16/19 | 40.7 | FSL | MNI | 3.0 T |
| 2015 | van de Pavert S.H.P. | PPMS | 25 | 11/14 | 52.5 | 12.0 |  | 30 | 12/18 | 37.8 | SPM8 | MNI | 3.0 T |
|  | van de Pavert S.H.P. | RRMS | 30 | 10/20 | 42.5 | 11.5 |  | 30 | 12/18 | 37.8 | SPM8 | MNI | 3.0 T |
|  | van de Pavert S.H.P. | SPMS | 25 | 11/14 | 52.8 | 24.0 |  | 30 | 12/18 | 37.8 | SPM8 | MNI | 3.0 T |
| 2016 | Sanchis-Seguraa C. | F-RRMS | 34 | 0/34 | 40.9 | 8.8 |  | 28 | 0/28 | 28.0 | SPM8 | MNI | 1.5 T |
|  | Sanchis-Seguraa C. | M-RRMS | 22 | 22/0 | 38.7 | 6.4 |  | 35 | 35/0 | 25.5 | SPM8 | MNI | 1.5 T |
| 2016 | Preziosa P. | CI-RRMS | 23 | 7/16 | 43.3 | 9.9 |  | 61 | 26/35 | 36.0 | SPM8 | MNI | 3.0 T |
|  | Preziosa P. | CP-RRMS | 38 | 14/24 | 37.5 | 7.1 |  | 61 | 26/35 | 36.0 | SPM8 | MNI | 3.0 T |
| 2016 | Zhang X. | RRMS | 39 | 16/23 | 38.3 | 7.7 |  | 29 | 12/17 | 37.8 | SPM8 | MNI | 3.0 T |
| 2017 | Dochea E. | RRMS | 23 | 4/19 | 34.2 | 4.5 |  | 16 | 4/12 | 37.1 | SPM8 | Talairach | 3.0 T |
| 2017 | Han X. | RRMS | 20 | 4/16 | 40.3 | - |  | 20 | 8/12 | 38.4 | SPM8 | MNI | 3.0 T |
| 2018 | Bisecco A. | RRMS | 125 | 43/82 | 36.8 | 9.6 |  | 52 | 19/33 | 37.3 | SPM12 | MNI | 3.0 T |
| 2019 | Campo C.G. | Fa-RRMS | 16 | 2/14 | 42.2 | 11.3 |  | 28 | 5/23 | 35.8 | SPM12 | MNI | 3.0 T |
|  | Campo C.G. | nFa-RRMS | 13 | 4/9 | 36.3 | 7.9 |  | 28 | 5/23 | 35.8 | SPM12 | MNI | 3.0 T |
| 2019 | Czekóová K. | RRMS | 43 | 12/31 | 35.8 | 7.5 |  | 43 | 18/25 | 34.7 | FSL | MNI | 1.5 T |
| 2020 | Emilie.L. | MS | 35 | 14/21 | 35 | 13 |  | 36 | 20/16 | - | SPM12 | MNI | 3.0 T |
| 2021 | Ornella Argento. | RRMS | 27 | - | 43.56 | 10.52 |  | 23 | - | 39.87 | SPM8 | MNI | 3.0 T |
|  | Ornella Argento. | SPMS | 16 | - | 43.56 | 10.52 |  | 23 | - | 39.87 | SPM8 | MNI | 3.0 T |
| 2021 | Eva Němá | RRMS | 51 | - | 31.81 | - |  | 42 | - | - | FSL | MNI | 1.5 T |
| 2021 | Hossein Shooli | RRMS | 19 | - | 35.9 | - |  | 14 | - | 34.8 | SPM | MNI | 1.5 T |
| 2022 | Elisabetta P. | RRMS | 466 | 323/143 | 37.3 | 10 |  | 279 | 164/115 | 38.2 | SPM12 | MNI | 3.0 T |
| 2023 | Giuseppe P. | RRMS | 117 | 32/85 | 40.6 | 12.7 |  | 53 | 33/20 | 41.3 | CAT12 | MNI | 3.0 T |

Abbreviation: BMS, benign form of multiple sclerosis; C, classical form of MS; Ce, cerebellar signs; CI, cognitively impaired; Cort, cortical form of multiple sclerosis; CP, cognitively preserved; F, female; Fa, fatigued; HC, healthy controls; M, male; MNI, Montreal Neurological Institute; MS, multiple sclerosis; nCe, no cerebellar signs; nFa, nonfatigued; PPMS, primary progressive multiple sclerosis; RRMS, relapsing-remitting multiple sclerosis; SPM, Statistical Parametric Mapping; SPMS, secondary progressive multiple sclerosis; T, tesla. ^a^ Mean.

**Table S3. Detailed demographic and clinical characteristics of the 50 datasets related to AD included in the meta-analysis.**

| Study | | AD | | | | | | | | | HC | | | Analysis parameter | | |
| --- | --- | --- | --- | --- | --- | --- | --- | --- | --- | --- | --- | --- | --- | --- | --- | --- |
| Year | Authors | Diagnosis | Disease stage | MMSE  (SD) | CDR  (SD) | Syndromic subtype | Sample size | M/F | Age^a^ (year) | Disease duration^a^  (year) | Sample size | M/F | Age^a^  (year) | Analysis software | Standard template | MRI field strength |
| 2001 | Baron J | AD | Mild AD | 22.4 ± 2.5 | - | - | 19 | - | 73 | - | 16 | - | 66 | SPM99 | Talairach | 1.5 T |
| 2002 | Frison G. | AD | Mild to moderate AD | 21 ± 4 | 1 | - | 29 | - | 74 | - | 26 | - | 69 | SPM99 | Talairach | 1.5 T |
| 2003 | Boxer A. | AD | - | 20.2 ± 7.3 | - | - | 11 | 8/3 | 69.6 | - | 15 | 7/8 | 65.1 | SPM99 | MNI | 3.0 T |
| 2003 | Busatto F. | AD | Mild AD | 20.7 ± 3.1 | - | - | 14 | 9/5 | 72.2 | - | 14 | 8/6 | 69.4 | SPM99 | Talairach | 1.5 T |
|  |  | AD | Mild AD | - | - | - | 11 | - | - | - | 14 | 8/6 | 69.4 | SPM99 | Talairach | 1.5 T |
| 2004 | Brenneis C. | AD | - | 17.4 ± 7.9 | - | - | 10 | 3/7 | 73.1 | - | 10 | 6/4 | 65.1 | SPM99 | Talairach | 1.5 T |
| 2005 | Zahn R. | AD | - | 23.6 ± 2.8 | - | - | 10 | 4/6 | 66.5 | - | 10 | 5/5 | 65.8 | SPM2 | MNI | 1.5 T |
| 2005 | Ishii K. | EO-AD | Mild AD | 23.0 ± 2.1 | - | - | 20 | 6/14 | 60.8 | - | 20 | 3/17 | 59.1 | SPM99 | Talairach | 1.5 T |
|  |  | LO-AD | Mild AD | 22.3 ± 1.8 | - | - | 20 | 3/17 | 72.2 | - | 20 | 9/11 | 70.3 | SPM99 | Talairach | 1.5 T |
| 2005 | Hirata Y. | AD | - | 26.0 ± 1.5 | 0.5 | - | 61 | 32/29 | 70.6 | - | 82 | 39/43 | 70.2 | SPM2 | Talairach | 1.0 T |
| 2006 | Baxter L. | AD | Mild to moderate AD | - | - | - | 15 | 11/4 | 75.5 | - | 15 | 7/8 | 76.4 | SPM2 | MNI | 1.5 T |
| 2006 | Bozzali M. | AD | - | 19.8 ± 4.1 | - | - | 22 | 11/11 | 67.9 | 24 | 20 | 13/7 | 65.8 | SPM2 | MNI | 1.5 T |
| 2006 | Xie S. | AD | Mild AD | 21.1 (19-24) | - | - | 13 | 8/5 | 71.7 | - | 16 | 10/6 | 71 | SPM2 | MNI | 1.5 T |
| 2007 | DiPaola M. | AD | - | 16.9 ± 4.3 | - | - | 18 | 4/14 | 64.3 | - | 18 | 4/14 | 65.4 | SPM2 | Talairach | 1.5 T |
| 2007 | Rabinovic. | AD | - | 19.9 ± 6.9 | 1.2 ± 0.5 | - | 11 | 5/6 | 64.5 | - | 40 | 17/23 | 63.5 | SPM2 | MNI | 1.5 T |
| 2007 | Whitwell J. | AD | - | 17.0 (13.0-24.0) | - | - | 38 | 16/22 | 65.3 | 4.7 | 38 | 16/22 | 65.9 | SPM2 | MNI | 1.5 T |
| 2007 | Hamalainen | AD | Mild AD | 21.7 ± 3.7 | - | - | 15 | - | 73.1 | - | 21 | - | 71.2 | SPM2 | MNI | 1.5 T |
| 2007 | Samuraki. | AD | Mild AD | 22.3 ± 3.3 | 1 | - | 39 | 19/20 | 68.3 | - | 73 | 37/36 | 66.78 | SPM2 | Talairach | 1.5 T |
| 2007 | Caroli | AD | - | 26.8 ± 1.8 | - | - | 9 | 5/4 | 69 | - | 17 | 8/9 | 69 | SPM2 | Talairach | - |
| 2007 | Matsunari | AD | Mild AD | 22.1 ± 3.5 | - | - | 27 | 12/15 | 68.6 | 2.4 | 40 | 20/20 | 66.5 | SPM2 | Talairach | 1.5 T |
| 2008 | Mazere | AD | Mild AD | 23.75 ± 1.58 | - | - | 8 | 3/5 | 80 | - | 8 | 2/6 | 73.8 | SPM2 | Talairach | 1.5 T |
| 2009 | Brambati | AD | Mild AD | 22.5 ± 2.3 | - | - | 10 | - | 71.5 | - | 13 | - | 75 | SPM5 | MNI | 3.0 T |
| 2009 | Honea | AD | - | 26.2 ± 3.7 | - | - | 60 | - | 74.3 | - | 56 | - | 73.3 | SPM5 | Talairach | 3.0 T |
| 2010 | Serra L. | AD | - | 18.2 ± 4.4 | - | - | 9 | 3/6 | 72.4 | - | 13 | 9/4 | 64.1 | SPM5 | MNI | 3.0 T |
| 2010 | Takahashi | AD | - | 18.7 ± 4.0 | - | - | 51 | 21/30 | 72.6 | - | 40 | 20/20 | 72 | SPM8 | Talairach | 1.5 T |
| 2011 | Gili T. | AD | - | 19.7 ± 4.5 | - | - | 11 | 7/4 | 71.9 | - | 10 | 7/3 | 64.1 | SPM5 | MNI | 3.0 T |
| 2011 | Lehmann M | Typical AD | - | 18.5 ± 4.6 | - | Amnestic AD | 30 | 14/16 | 69.2 | 5.4 | 50 | 17/33 | 63.7 | SPM5 | MNI | 1.5 T |
| 2011 | Dashjamts | AD | - | 21.1 ± 4.4 | - | - | 23 | 9/14 | 74.6 | - | 23 | 11/12 | 73.2 | SPM8 | Talairach | 3.0 T |
| 2011 | Dos Santos | AD | Mild AD | 21.41 ± 2.17 | 0.82 ± 0.24 | - | 34 | 23/11 | 70.3 | - | 32 | 17/15 | 68.7 | SPM2 | MNI | 1.5 T |
| 2011 | Whitwell | Typical-AD | - | 18.8 ± 6.1 | - | Amnestic AD | 14 | 7/7 | 67.4 | - | 20 | 11/9 | 68.7 | SPM2 | MNI | 1.5 T |
|  |  | Atypical-AD | - | 22.8 ± 5.5 | - | - | 14 | 7/7 | 67.2 | - | 20 | 11/9 | 68.7 | SPM2 | MNI | 1.5 T |
| 2012 | Rami. L | AD | Mild AD | 22.5 ± 3.3 | - | - | 32 | - | 75.5 | - | 24 | - | 71.4 | SPM5 | MNI | 3.0 T |
|  |  | Prd-AD | - | 24.8 ± 1.6 | - | - | 14 | - | 71.6 | - | 24 | - | 71.4 | SPM5 | MNI | 3.0 T |
| 2012 | Teipel. S | AD | Mild to moderate AD | 20.6 ± 5.3 | - | - | 137 | - | 72.5 | - | 143 | - | 69.2 | SPM8 | Talairach | 3.0 T |
| 2012 | Bozzali. M | AD | - | 17.8 ± 4.2 | - | - | 31 | 12/19 | 72.8 | - | 14 | 10/4 | 68 | SPM8 | MNI | 3.0 T |
| 2012 | Tondelli. M | AD | - | 27.62 ± 1.05 | - | - | 8 | 2/6 | 79.4 | - | 40 | 20/20 | 76.1 | FSL | MNI | 1.5 T |
| 2013 | Imabayashi | AD | - | - | - | - | 5 | 2/3 | 73.8 | - | 7 | 4/3 | 70.1 | SPM8 | Talairach | 1.5 T |
| 2014 | Guo. Y | AD | Mild AD | 19.7 ± 4.1 | 1.3 ± 0.5 | - | 35 | 12/23 | 72.4 | - | 27 | 16/11 | 69.2 | SPM8 | MNI | - |
| 2016 | Dermody | AD | - | - | - | - | 25 | 15/10 | 66.1 | - | 22 | 13/9 | 68.2 | FSL | MNI | 3.0 T |
| 2017 | Roquet | AD | - | 27.1 ± 1.4 | - | - | 28 | 9/19 | 74.1 | - | 22 | 10/12 | 65.6 | SPM8 | MNI | 3.0 T |
| 2017 | Tascone | fNPS-AD | Mild to moderate AD | 15.14 ± 4.49 | 0.5-2.0 | - | 7 | 1/6 | 72.2 | - | 13 | 2/11 | 69.5 | SPM8 | MNI | 1.5 T |
|  |  | mNPS-AD | Mild to moderate AD | 18.42 ± 5.35 | 0.5-2.0 | - | 12 | 4/8 | 74 | - | 13 | 2/11 | 69.5 | SPM8 | MNI | 1.5 T |
| 2018 | Jia. X | AD | - | 16.30 ± 6.04 | 1.11 ± 0.57 | - | 50 | 22/28 | 71.3 | - | 50 | - | 70.16 | SPM8 | MNI | 3.0 T |
| 2019 | Kunst. J | AD | - | 20.9 ± 2.3 | - | - | 12 | 8/4 | 72.9 | - | 58 | 31/27 | 67.5 | SPM12 | MNI | - |
| 2020 | G. Fumagalli | Typical AD | - | 19.55 ± 5.3 | - | - | 30 | 8/22 | 68.93 | - | 15 | 7/8 | 69.51 | SMP12 | MNI | 3.0 T |
| 2020 | Hiscox. L | AD | Mild to moderate AD | - | - | Amnestic AD | 12 | 5/7 | 77.2 | - | 12 | 6/6 | 69.4 | SPM12 | MNI | 3.0 T |
| 2020 | Y. Wu | AD | - | 15.55 ± 3.99 | 1.15 ± 0.54 | - | 20 | 11/9 | 67.15 | - | 22 | 13/9 | 68.41 | VBN8 | MNI | 3.0 T |
| 2021 | Shaw.S. | AD | - | - | - | - | 30 | 16/14 | 66.2 | 4.8 | 30 | 15/15 | 66.2 | FSL | MNI | 3.0 T |
| 2021 | Caso. F | AD | Mild to moderate AD | 17.5 ± 2.5 | 1.4 ± 0.5 | - | 26 | 7/19 | 71.1 | - | 20 | 9/11 | 69.6 | SPM12 | MNI | 3.0 T |
| 2021 | van de Mortel | AD | - | 23.1 ± 2.02 | 0.5-1.0 | - | 239 | 117/122 | 75.4 | - | 20 | 9/11 | 69.6 | SPM12 | MNI | 3.0 T |
| 2021 | A. Birba | AD | - | - | 1.14 ± 0.65 | - | 23 | 7/16 | 73.3 | - | 41 | 11/30 | 72.05 | SPM12 | - | - |
| 2022 | P.He | AD | - | 16.56 ± 4.25 | 1 | - | 16 | 8/8 | 70.68 |  | 30 | 15/15 | 64.63 | SPM12 | MNI | 3.0 T |
| 2023 | Ke Wan | EO-AD | - | 23.2 ± 2.9 | 0.6 ± 0.2 | - | 47 | 17/30 | 65 | - | 52 | 30/22 | 62.5 | SPM8 | MNI | 3.0 T |
| 2024 | C. Saha | AD | - | - | 1.1 ± 0.3 | - | 83 | 44/39 | 73.1 | - | 85 | 47/38 | 74.4 | SPM12 | MNI | 3.0 T |
| 2024 | L. Frings | AD | - | 23 (21-25) | - | - | 26 | 14/12 | 62.6 | - | 13 | 7/6 | 68.3 | SPM12 | MNI | 3.0 T |

Abbreviation: AD, Alzheimer's disease; CDR, clinical dementia rating; EO, early onset; F, female; fNPS, few neuropsychiatric symptoms; HC, healthy controls; LO, late-onset; M, male; MMSE, mini‑mental state examination; MNI, Montreal Neurological Institute; mNPS, multiple neuropsychiatric symptoms; PrdAD, prodromal AD patients; SPM, Statistical Parametric Mapping; T, tesla. ^a^,Me

**Table S4. FWE-corrected sensitivity analysis of grey matter atrophy in MS compared with HC.**

| **Maximum Peak** | | | | | | |
| --- | --- | --- | --- | --- | --- | --- |
| **MNI Coordinates** | | | **SDM-Z** | ***P*** | **Voxels** | **Brain** |
| (x | y | z) | **value** | **value** | **Size** | **Regions** |
| 12 | -28 | 2 | -11.234 | 1.00E-03 | 721 | Right thalamus |
| -6 | -22 | 46 | -9.162 | 1.00E-03 | 1596 | Left posterior cingulate |
| -14 | 18 | 6 | -8.925 | 1.00E-03 | 315 | Left caudate |
| 14 | 12 | 10 | -8.916 | 1.00E-03 | 251 | Right caudate |
| -16 | -30 | 6 | -8.688 | 1.00E-03 | 502 | Left thalamus |
| 52 | -20 | 12 | -8.140 | 1.00E-03 | 3337 | Right supramarginal gyrus |
| 40 | -20 | 46 | -8.059 | 1.00E-03 | 1257 | Right postcentral gyrus |
| -46 | -10 | -2 | -7.880 | 1.00E-03 | 3213 | Left insula |
| -38 | -24 | 48 | -6.821 | 1.00E-03 | 727 | Left postcentral gyrus |

*Abbreviation: MS, multiple sclerosis; FWE, family-wise error;* *HC, healthy control; MNI, Montreal Neurological Institute.*

**Table S5. FWE-corrected sensitivity analysis of grey matter atrophy in AD compared with HC.**

| **Maximum Peak** | | | | | | |
| --- | --- | --- | --- | --- | --- | --- |
| **MNI Coordinates** | | | **SDM-Z** | ***P*** | **Voxels** | **Brain** |
| (x | y | z) | **value** | **value** | **Size** | **Regions** |
| 20 | -2 | -22 | -14.153 | 1.00E-03 | 7766 | Right parahippocampal gyrus |
| -24 | -10 | -24 | -12.971 | 1.00E-03 | 4736 | Left hippocampus |
| -56 | -54 | 20 | -8.698 | 1.00E-03 | 1351 | Left angular gyrus |

*Abbreviation: AD, multiple sclerosis; FWE, family-wise error; HC, healthy control; MNI, Montreal Neurological Institute.*

**Table S6. Egger’s test and Jackknife test of each peak in MS and HC subgroup meta-analysis.**

| Peak | Bias | Z-score | Degree of freedom | *P* value | Jackknife test |
| --- | --- | --- | --- | --- | --- |
| 1 | -0.21 | -0.26 | 32 | 0.798 | 33/34 |
| 2 | 0.19 | 0.39 | 32 | 0.696 | 34/34 |
| 3 | 0.03 | 0.08 | 32 | 0.939 | 33/34 |
| 4 | -0.28 | -0.53 | 32 | 0.596 | 33/34 |
| 5 | -0.16 | -0.28 | 32 | 0.777 | 31/34 |
| 6 | 0.23 | 0.44 | 32 | 0.657 | 30/34 |
| 7 | 0.46 | 0.68 | 32 | 0.495 | 33/34 |
| 8 | 0.52 | 1.09 | 32 | 0.277 | 33/34 |
| 9 | 0.20 | 0.40 | 32 | 0.689 | 33/34 |

Abbreviation: MS, multiple sclerosis; HC, healthy controls.

**Table S7. Egger’s test and Jackknife test of each peak in AD and HC subgroup meta-analysis.**

| Peak | Bias | Z-score | Degree of freedom | *P* value | Jackknife test |
| --- | --- | --- | --- | --- | --- |
| 1 | -0.31 | -0.63 | 48 | 0.531 | 49/50 |
| 2 | -0.08 | -0.17 | 48 | 0.866 | 45/50 |
| 3 | -0.27 | -0.55 | 48 | 0.582 | 49/50 |
| 4 | -0.26 | -0.39 | 48 | 0.695 | 47/50 |

Abbreviation: AD, Alzheimer's disease; HC, healthy controls.

**Table S8. Age-adjusted meta-regression results for grey matter differences between MS and AD.**

| **Maximum Peak** | | | | | | |
| --- | --- | --- | --- | --- | --- | --- |
| **MNI Coordinates** | | | **SDM-Z** | ***P*** | **Voxels** | **Brain** |
| (x | y | z) | **value** | **value** | **Size** | **Regions** |
| MS < AD | | | | | | |
| 48 | -16 | 46 | -3.933 | 4.20E-05 | 267 | Right precentral gyrus |
| -48 | -18 | 52 | -4.087 | 2.18E-05 | 54 | Left postcentral gyrus |
| 38 | -26 | 58 | -3.215 | 6.53E-04 | 33 | Right precentral gyrus |
| AD < MS | | | | | | |
| -54 | -54 | 28 | -3.952 | 3.87E-05 | 84 | Left supramarginal gyrus |

**Table S9. Enrichment terms of the BP related to common positive gene of MS and AD.**

| Source | Term | *P* value |
| --- | --- | --- |
| GO:BP | oxidation-reduction process | 0.000999 |
| GO:BP | DNA damage checkpoint | 0.000999 |
| GO:BP | glucose metabolic process | 0.000999 |
| GO:BP | glucose transmembrane transport | 0.000999 |
| GO:BP | meiotic cell cycle | 0.000999 |
| GO:BP | mitophagy | 0.001998 |
| GO:BP | response to organonitrogen compound | 0.002997 |
| GO:BP | glycogen catabolic process | 0.002997 |
| GO:BP | cell cycle arrest | 0.003996 |
| GO:BP | telomere maintenance | 0.005994 |
| GO:BP | DNA replication | 0.006993 |
| GO:BP | DNA damage response | 0.006993 |
| GO:BP | intrinsic apoptotic signaling pathway | 0.006993 |
| GO:BP | B cell differentiation | 0.006993 |
| GO:BP | T cell receptor signaling pathway | 0.006993 |
| GO:BP | macroautophagy | 0.002997 |
| GO:BP | double-strand break repair via nonhomologous end joining | 0.006993 |
| GO:BP | mitotic cell cycle checkpoint | 0.006993 |
| GO:BP | mitotic G2 DNA damage checkpoint | 0.006993 |
| GO:BP | regulation of DNA-dependent DNA replication initiation | 0.006993 |
| GO:BP | blastocyst growth | 0.006993 |
| GO:BP | DNA double-strand break processing | 0.006993 |
| GO:BP | double-strand break repair | 0.006993 |
| GO:BP | positive regulation of telomere maintenance | 0.006993 |
| GO:BP | DNA duplex unwinding | 0.006993 |
| GO:BP | positive regulation of kinase activity | 0.006993 |
| GO:BP | isotype switching | 0.006993 |
| GO:BP | t-circle formation | 0.006993 |
| GO:BP | telomeric 3 overhang formation | 0.006993 |
| GO:BP | negative regulation of telomere capping | 0.006993 |
| GO:BP | neuronal stem cell population maintenance | 0.008991 |
| GO:BP | cellular response to DNA damage stimulus | 0.010989 |
| GO:BP | transmembrane transport | 0.010989 |
| GO:BP | cellular protein catabolic process | 0.013986 |
| GO:BP | neuromuscular process controlling balance | 0.013986 |
| GO:BP | protein K63-linked ubiquitination | 0.013986 |
| GO:BP | endoderm development | 0.017982 |
| GO:BP | anatomical structure morphogenesis | 0.017982 |
| GO:BP | positive regulation of protein autophosphorylation | 0.017982 |
| GO:BP | spleen development | 0.017982 |
| GO:BP | retinal ganglion cell axon guidance | 0.021978 |
| GO:BP | myo-inositol transport | 0.022977 |
| GO:BP | riboflavin metabolic process | 0.023976 |
| GO:BP | riboflavin biosynthetic process | 0.023976 |
| GO:BP | FMN biosynthetic process | 0.023976 |
| GO:BP | positive regulation of NAD(P)H oxidase activity | 0.023976 |
| GO:BP | steroid hormone mediated signaling pathway | 0.023976 |
| GO:BP | positive regulation of NIK/NF-kappaB signaling | 0.023976 |
| GO:BP | negative regulation of cell proliferation | 0.025974 |
| GO:BP | CMP salvage | 0.027972 |
| GO:BP | UMP salvage | 0.027972 |
| GO:BP | CTP salvage | 0.027972 |
| GO:BP | activation of MAPKK activity | 0.02997 |
| GO:BP | activation of MAPK activity | 0.02997 |
| GO:BP | positive regulation of T cell cytokine production | 0.02997 |
| GO:BP | MyD88-dependent toll-like receptor signaling pathway | 0.02997 |
| GO:BP | Wnt signaling pathway, calcium modulating pathway | 0.02997 |
| GO:BP | I-kappaB kinase/NF-kappaB signaling | 0.02997 |
| GO:BP | activation of NF-kappaB-inducing kinase activity | 0.02997 |
| GO:BP | I-kappaB phosphorylation | 0.02997 |
| GO:BP | positive regulation of macroautophagy | 0.02997 |
| GO:BP | positive regulation of interleukin-2 production | 0.02997 |
| GO:BP | positive regulation of I-kappaB kinase/NF-kappaB signaling | 0.02997 |
| GO:BP | anoikis | 0.02997 |
| GO:BP | positive regulation of JUN kinase activity | 0.02997 |
| GO:BP | histone H3 acetylation | 0.02997 |
| GO:BP | regulation of respiratory gaseous exchange | 0.02997 |
| GO:BP | positive regulation of NF-kappaB transcription factor | 0.02997 |
| GO:BP | stress-activated MAPK cascade | 0.02997 |
| GO:BP | nucleotide-binding oligomerization domain | 0.02997 |
| GO:BP | interleukin-1-mediated signaling pathway | 0.02997 |
| GO:BP | kidney development | 0.030969 |
| GO:BP | renal system process | 0.030969 |
| GO:BP | mitochondrial DNA replication | 0.030969 |
| GO:BP | DNA repair | 0.030969 |
| GO:BP | deoxyribonucleoside triphosphate metabolic process | 0.030969 |
| GO:BP | response to amine | 0.030969 |
| GO:BP | nucleobase-containing small molecule interconversion | 0.030969 |
| GO:BP | intrinsic apoptotic signaling pathway by p53 class mediator | 0.030969 |
| GO:BP | regulation of signal transduction by p53 class mediator | 0.034965 |
| GO:BP | NLS-bearing protein import into nucleus | 0.035964 |
| GO:BP | modulation by virus of host process | 0.035964 |
| GO:BP | viral penetration into host nucleus | 0.035964 |
| GO:BP | regulation of protein localization | 0.03996 |
| GO:BP | protein autoubiquitination | 0.03996 |
| GO:BP | mitochondrial protein catabolic process | 0.043956 |
| GO:BP | mitophagy by induced vacuole formation | 0.043956 |
| GO:BP | positive regulation of protein phosphorylation | 0.046953 |
| GO:BP | myeloid progenitor cell differentiation | 0.047952 |

Abbreviation: AD, Alzheimer's disease; BP, biological process; MS, multiple sclerosis.

**Table S10. Enrichment terms of the BP related to common negative gene of MS and AD.**

| Source | Term | *P* value |
| --- | --- | --- |
| GO:BP | histone H3-K9 methylation | 0.000999 |
| GO:BP | response to vitamin | 0.000999 |
| GO:BP | mitotic cell cycle | 0.000999 |
| GO:BP | histamine secretion | 0.000999 |
| GO:BP | negative regulation of acute inflammatory response | 0.000999 |
| GO:BP | regulation of interleukin-1 beta production | 0.000999 |
| GO:BP | negative regulation of interferon-beta production | 0.000999 |
| GO:BP | negative regulation of interleukin-6 production | 0.002997 |
| GO:BP | follicular dendritic cell differentiation | 0.003996 |
| GO:BP | marginal zone B cell differentiation | 0.003996 |
| GO:BP | positive regulation of methylation-dependent chromatin silencing | 0.003996 |
| GO:BP | Synaptic vesicle transport | 0.003996 |
| GO:BP | DNA replication | 0.003996 |
| GO:BP | B cell differentiation | 0.003996 |
| GO:BP | deoxyribonucleoside catabolic process | 0.003996 |
| GO:BP | placenta blood vessel development | 0.003996 |
| GO:BP | trophoblast giant cell differentiation | 0.003996 |
| GO:BP | regulation of transcription involved in lymphatic endothelial cell | 0.003996 |
| GO:BP | phospholipid biosynthetic process | 0.004995 |
| GO:BP | radial pattern formation | 0.004995 |
| GO:BP | regulation of tumor necrosis factor production | 0.008991 |
| GO:BP | negative regulation of acute inflammatory response | 0.008991 |
| GO:BP | negative regulation of muscle cell apoptotic process | 0.008991 |
| GO:BP | response to starvation | 0.008991 |
| GO:BP | negative regulation of potassium ion transport | 0.008991 |
| GO:BP | skeletal muscle tissue | 0.008991 |
| GO:BP | positive regulation of translation | 0.008991 |
| GO:BP | positive regulation of growth hormone secretion | 0.008991 |
| GO:BP | negative regulation of glial cell proliferation | 0.008991 |
| GO:BP | regulation of oligodendrocyte progenitor proliferation | 0.008991 |
| GO:BP | positive regulation of chemokine (C-C motif) ligand 5 production | 0.008991 |
| GO:BP | positive regulation of somatostatin secretion | 0.008991 |
| GO:BP | fertilization | 0.010989 |
| GO:BP | cAMP-mediated signaling | 0.010989 |
| GO:BP | regulation of actin polymerization or depolymerization | 0.015984 |
| GO:BP | positive regulation of cytokinesis | 0.015984 |
| GO:BP | regulation of protein localization | 0.016983 |
| GO:BP | germinal center formation | 0.018981 |
| GO:BP | activation of adenylate cyclase activity | 0.018981 |
| GO:BP | activation of transmembrane receptor protein tyrosine kinase activity | 0.01998 |
| GO:BP | maternal placenta development | 0.01998 |
| GO:BP | regulation of cell-cell adhesion | 0.01998 |
| GO:BP | regulation of cell morphogenesis | 0.01998 |
| GO:BP | regulation of actin cytoskeleton organization | 0.01998 |
| GO:BP | collateral sprouting | 0.01998 |
| GO:BP | positive regulation of collateral sprouting | 0.01998 |
| GO:BP | negative chemotaxis | 0.01998 |
| GO:BP | regulation of focal adhesion assembly | 0.01998 |
| GO:BP | regulation of insulin secretion involved in cellular r | 0.01998 |
| GO:BP | cellular response to forskolin | 0.01998 |
| GO:BP | positive regulation of cell-substrate adhesion | 0.021978 |
| GO:BP | negative regulation of BMP signaling pathway | 0.021978 |
| GO:BP | positive regulation of cell cycle | 0.022977 |
| GO:BP | spinal cord motor neuron differentiation | 0.023976 |
| GO:BP | positive regulation of systemic arterial blood pressure | 0.023976 |
| GO:BP | humoral immune response mediated by circulating immunoglobulin | 0.023976 |
| GO:BP | response to UV-C | 0.023976 |
| GO:BP | antimicrobial humoral response | 0.023976 |
| GO:BP | DNA damage response, signal transduction by p53 class mediator | 0.023976 |
| GO:BP | positive regulation of interferon-gamma production | 0.023976 |
| GO:BP | T-helper 1 type immune response | 0.023976 |
| GO:BP | negative regulation of tumor necrosis factor biosynthetic process | 0.023976 |
| GO:BP | intrinsic apoptotic signaling pathway in response to DNA damage | 0.023976 |
| GO:BP | T-helper 2 cell differentiation | 0.023976 |
| GO:BP | positive regulation of interleukin-10 biosynthetic process | 0.023976 |
| GO:BP | negative regulation of interleukin-8 biosynthetic process | 0.023976 |
| GO:BP | negative regulation of JAK-STAT cascade | 0.023976 |
| GO:BP | regulation of DNA binding | 0.023976 |
| GO:BP | maintenance of protein location in nucleus | 0.023976 |
| GO:BP | regulation of NIK/NF-kappaB signaling | 0.023976 |
| GO:BP | negative regulation of chloride transport | 0.023976 |
| GO:BP | unsaturated fatty acid biosynthetic process | 0.024975 |
| GO:BP | exocytosis | 0.024975 |
| GO:BP | alpha-linolenic acid metabolic process | 0.024975 |
| GO:BP | protein processing | 0.028971 |
| GO:BP | negative regulation of cytokine production | 0.030969 |
| GO:BP | regulation of protein kinase activity | 0.031968 |
| GO:BP | epithelial fluid transport | 0.032967 |
| GO:BP | inositol phosphate-mediated signaling | 0.032967 |
| GO:BP | negative regulation of calcium-mediated signaling | 0.032967 |
| GO:BP | regulation of postsynaptic cytosolic calcium ion concentration | 0.032967 |
| GO:BP | steroid hormone mediated signaling pathway | 0.034965 |
| GO:BP | regulation of microtubule cytoskeleton organization | 0.037962 |
| GO:BP | inner ear development | 0.042957 |
| GO:BP | pentose-phosphate shunt | 0.043956 |
| GO:BP | neuron recognition | 0.043956 |
| GO:BP | positive regulation of cAMP-mediated signaling | 0.043956 |
| GO:BP | deoxyribose phosphate catabolic process | 0.043956 |
| GO:BP | negative regulation of JNK cascade | 0.044955 |
| GO:BP | negative regulation of single stranded | 0.046953 |
| GO:BP | negative regulation of protein phosphorylation | 0.04995 |
| GO:BP | platelet activation | 0.04995 |
| GO:BP | negative regulation of endothelial cell migration | 0.04995 |
| GO:BP | positive regulation of glucose import | 0.04995 |
| GO:BP | negative regulation of cyclin-dependent protein serine | 0.04995 |
| GO:BP | generation of neurons | 0.04995 |
| GO:BP | negative regulation of phagocytosis | 0.04995 |
| GO:BP | cellular response to interleukin-12 | 0.04995 |
| GO:BP | negative regulation of macrophage inflammatory protein 1 alpha production | 0.04995 |
| GO:BP | negative regulation of chemokine (C-C motif) ligand 5 production | 0.04995 |
| GO:BP | negative regulation of I-kappaB phosphorylation | 0.04995 |

Abbreviation: AD, Alzheimer's disease; BP, biological process; MS, multiple sclerosis.

**Table S11. The cell types that PLS2+ gene sets were expressed derived from specific expression analysis.**

| Brain cell types | Uncorrected *p*-values | Gene number |
| --- | --- | --- |
| **Striatum (caudate and putamen)** | 0.001 | 12 |
| Drd2+ medium spiny neurons of striatum | 0.008 | 15 |
| Drd1+ medium spiny neurons of striatum | 0.012 | 16 |

*Note:* Fisher's exact test with Benjamini-Hochberg (*q* < 0.05) was utilized, and highlighted in bold.

**Table S12. The cell types that PLS2- gene sets were expressed derived from specific expression analysis**

| Brain cell types | Uncorrected *p*-values | Gene number |
| --- | --- | --- |
| **Immune Cells** | 0.00000158 | 26 |
| **Pnoc+ neurons of cortex** | 0.0007077 | 29 |
| **Habenula (epithalamus)** | 0.0009017 | 13 |
| **Hypothalamus** | 0.002 | 17 |
| **Cholinergic neurons of striatum** | 0.006 | 16 |
| Serotonergic neurons of brain stem | 0.014 | 9 |
| Ntsr+ neurons of cortex | 0.017 | 15 |
| Cortex | 0.02 | 13 |
| Cholinergic neurons of habenula | 0.026 | 10 |
| Cholinergic neurons of basal forebrain | 0.029 | 12 |

*Note:* Fisher's exact test with Benjamini-Hochberg (*q* < 0.05) was utilized, and highlighted in bold.

**3. Supplementary Figures**

**Figure S1.** A scatterplot of the correlation between the genes in component 2 and common patterns of GMV reduction in MS and AD. The x-axis shows the PLS scores of component 2, and the y-axis shows the *Z* values of the shared GMV map in MS and AD. The PLS scores of component 2 are positive correlated with the common patterns (*Spearman*'s *r* = 0.37, *P* = 0.001, permutation test with SA corrected, 1000 permutations).

Abbreviation: PLS2, the second component of the partial least squares regression

***Figure S2. KEGG pathway enrichment analysis of positive- and negative-weighted genes.*** *Circles represent the positive-weighted gene set, whereas triangles represent the negative-weighted gene set. The x-axis indicates the percentage of genes enriched in each KEGG pathway. Symbol size represents the number of genes involved in each pathway, and color represents the -log10(FDR-adjusted q value), with warmer colors indicating greater statistical significance.*

**4. References**

1.	Arnatkeviciute, A., Fulcher, B. D. & Fornito, A. (2019)A practical guide to linking brain-wide gene expression and neuroimaging data. Neuroimage 189. 353-367.<https://doi.org/10.1016/j.neuroimage.2019.01.011>

2.	Hawrylycz, M. *et al.* (2015)Canonical genetic signatures of the adult human brain. Nat Neurosci 18. 1832-1844.<https://doi.org/10.1038/nn.4171>

3.	Hawrylycz, M. J. *et al.* (2012)An anatomically comprehensive atlas of the adult human brain transcriptome. Nature 489. 391-399.<https://doi.org/10.1038/nature11405>

4.	Markello, R. D. *et al.* (2021)Standardizing workflows in imaging transcriptomics with the abagen toolbox. Elife 10.<https://doi.org/10.7554/eLife.72129>
